# The epidemiology of travel-related *Salmonella* Enteritidis in Ontario, Canada, 2010–2011

**DOI:** 10.1186/1471-2458-12-310

**Published:** 2012-05-18

**Authors:** Mary-Kathryn Tighe, Rachel Savage, Linda Vrbova, Miriam Toolan, Yvonne Whitfield, Csaba Varga, Brenda Lee, Vanessa Allen, Anne Maki, Ryan Walton, Caitlin Johnson, Badal Dhar, Rafiq Ahmed, Natasha S Crowcroft, Dean Middleton

**Affiliations:** 1 Public Health Ontario, 480 University Ave, Toronto, M5G 1V2, Canada; 2 Canadian Field Epidemiology Program, Public Health Agency of Canada, 8 Colonnade Road, Ottawa, Ontario, K2E 7M6, Canada; 3 Guy’s, King’s and St Thomas’ School of Medicine, London, SE1 9RT, UK; 4 School of Population and Public Health, University of British Columbia, 2206 East Mall, Vancouver, BC, V6T 1Z3, Canada; 5 Ontario Ministry of Health and Long-Term Care, 1075 Bay St, Toronto, Ontario, M5S 2B1, Canada; 6 Department of Population Medicine, University of Guelph, Guelph, Ontario, N1G 2 W1, Canada; 7 Dalla Lana School of Public Health, University of Toronto, 155 College St., Health Sciences Building, 6th Floor, Toronto, Ontario, M5T 3 M7, Canada; 8 Laboratory Medicine and Pathobiology, University of Toronto, Toronto, Canada; 9 National Microbiology Laboratory, Public Health Agency of Canada, Ottawa, Canada

## Abstract

**Background:**

Increases in the number of salmonellosis cases due to *Salmonella* Enteritidis (SE) in 2010 and 2011 prompted a public health investigation in Ontario, Canada. In this report, we describe the current epidemiology of travel-related (TR) SE, compare demographics, symptoms and phage types (PTs) of TR and domestically-acquired (DA) cases, and estimate the odds of acquiring SE by region of the world visited.

**Methods:**

All incident cases of culture confirmed SE in Ontario obtained from isolates and specimens submitted to public health laboratories were included in this study. Demographic and illness characteristics of TR and DA cases were compared. A national travel survey was used to provide estimates for the number of travellers to various destinations to approximate rates of SE in travellers. Multivariate logistic regression was used to estimate the odds of acquiring SE when travelling to various world regions.

**Results:**

Overall, 51.9% of SE cases were TR during the study period. This ranged from 35.7% TR cases in the summer travel period to 65.1% TR cases in the winter travel period. Compared to DA cases, TR cases were older and were less likely to seek hospital care. For Ontario travellers, the adjusted odds of acquiring SE was the highest for the Caribbean (OR 37.29, 95% CI 17.87-77.82) when compared to Europe. Certain PTs were more commonly associated with travel (e.g., 1, 4, 5b, 7a, Atypical) than with domestic infection. Of the TR cases, 88.9% were associated with travel to the Caribbean and Mexico region, of whom 90.1% reported staying on a resort. Within this region, there were distinct associations between PTs and countries.

**Conclusions:**

There is a large burden of TR illness from SE in Ontario. Accurate classification of cases by travel history is important to better understand the source of infections. The findings emphasize the need to make travellers, especially to the Caribbean, and health professionals who provide advice to travellers, aware of this risk. The findings may be generalized to other jurisdictions with travel behaviours in their residents similar to Ontario residents.

## Background

*Salmonella* bacteria are estimated to cause 93.8 million cases of gastroenteritis and 155,000 deaths worldwide each year, with 2,800 of these deaths estimated to occur in North America [1]. In the last decade, the rate of reported cases of *Salmonella enterica* subsp. *enterica* serovar Enteritidis (SE) has increased steadily in Ontario. For the years 2000 to 2004, the average annual number of SE cases was 502 [2-5]. Following a large outbreak of SE in 2005, the average annual number was 710 cases for the years 2006 to 2009 [6-8]. In 2010, the number increased further to 1,035 cases, which prompted the investigation of SE illnesses in the province of Ontario [9]. A hypothesis-generating study was undertaken which identified several food items that were subsequently tested with a separate, unpublished case–control study.

In 2006, the estimated mean cost per case of gastroenteritis in Ontario was CAN $1,089 and the mean annual cost per capita was CAN $115 [10]. This represents a substantial and preventable burden of illness as well as a cost to the health care system. While surveillance of possible sources of domestically-acquired (DA) foodborne infection is standard practice, less is known about the epidemiology of travel-related (TR) enteric pathogens, and in particular TR SE. Variable proportions of cases of reported salmonellosis associated with international travel have been documented in several studies. Two Canadian studies estimated that the minority of *Salmonella* cases, ranging from 27% to 38%, were due to travel [11,12]. Another study from the United States (USA) identified 11% of *Salmonella* cases to be TR [13]. The significance of TR SE has been noted in Europe with a Danish study finding 25% of cases of SE to be TR between 1997 and 1999, and a Swedish study attributing 78% of *Salmonella* with TR status between 1997 and 2003 [14,15]. The number of TR cases by region or country of travel, without appropriate denominator information on travel patterns, may be more reflective of travel patterns than risk. Therefore, in order to assess the risk of SE associated with travel to different countries and regions, we used information collected during the hypothesis-generating study described above and subsequent case control study, along with a population-based travel survey to obtain denominator information. In this report, we compare demographics, symptoms and phage types (PTs) of TR and DA cases in order to describe the current epidemiology of TR SE in Ontario residents. Further, we estimate the risk of acquiring SE by region of the world visited.

## Methods

### Setting

Ontario is Canada’s largest province, consisting of 13.2 million residents in 2010 [16]. Ontario’s public health system consists of 36 health units responsible for the delivery of local public health programs, as well as one central and 10 regional public health laboratories operated by Public Health Ontario.

### Laboratory testing and methods

The public health laboratories serve as the reference centre for enteric bacteria in Ontario. The services provided include the primary isolation of enteric pathogens to support outbreak investigations, as well as reference identification, serotyping and molecular typing of enteric pathogens submitted from hospital and private laboratories. *Salmonella spp*. isolates from across the province are sent to the public health laboratories for serotyping based on the Kauffmann –White scheme [17]. As per routine practice, all isolates identified as SE are forwarded to the National Microbiology Laboratory in Winnipeg for phage typing using methods described by Ward and colleagues [18]. The laboratory and PT results are compiled daily at the Toronto public health laboratory and shared with the investigators in a line list format.

### Case ascertainment and data collection

We interviewed all incident cases with culture confirmed SE submitted to the Toronto public health laboratory during two investigation stages: a hypothesis-generating stage from July 11^th^ to November 30^th^, 2010 and a case–control study stage from January 20^th^ to July 31^st^, 2011. (The case–control study is a separate, unpublished study not described here. The purpose of the case–control study was to identify risk factors for DA cases. The controls used in the case–control study were healthy controls randomly selected from the Ontario population). Cases were excluded who: resided outside of Ontario or on a First Nations reserve; had SE isolated from a clinical specimen other than stool; had testing performed more than two months following symptom onset; or could not speak English. Additionally, only the first specimen was included for cases that had repeated specimens purposely performed. For this study, we also excluded cases with a symptom onset date prior to July 2010, during December 2010, and after June 2011. Cases were lost to follow-up if they died, did not have a telephone number available, or could not be reached following five attempts. Refusals were defined as those cases who declined to be interviewed.

We performed telephone interviews using two standardized questionnaires (one for each study stage) that collected demographic, clinical symptom, and travel information in a similar manner. The questionnaires also collected animal contact, food exposure, and food-hygiene practice information that was modified between the hypothesis-generating and case–control study stage based on the hypotheses generated. Parents or guardians responded on behalf of children less than 16 years of age. All respondents gave informed verbal consent prior to beginning the interview. Ethical approval was not required to interview cases because this was a public health investigation under the *Ontario Health Protection and Promotion Act*[19]. Data from questionnaires were entered into EpiData version 3.1 (Lauritsen JM & Bruus M. EpiData Entry).

A TR case was defined as an Ontario resident, with culture confirmed SE and a symptom onset date between July 2010 and June 2011 (excluding December 2010), who travelled outside of Canada within the three days prior to onset of illness. A DA case was defined as an Ontario resident, with culture confirmed SE identified and a symptom onset date between July 2010 and June 2011 (excluding December 2010), who did not travel outside of Canada in the three days prior to onset of illness. The time period from July to October 2010 and May to June 2011 was classified as the “summer travel period”, and the month of November 2010 and the time period from January to April 2011 were classified as the “winter travel period”. In order to generate total Ontario SE case counts by symptom onset month, we calculated an approximate symptom onset date for cases who were lost to follow-up using the date their sample was received at the laboratory minus the average number of days from symptom onset date to the date received at the laboratory for interviewed cases. The percent of travel cases by month was calculated by dividing the number of TR cases by the number of interviewed cases per month.

### Comparison of travel-related (TR) and domestically-acquired (DA) cases

TR and DA cases were compared to determine differences in demographics, health-seeking behaviour and illness characteristics using Wilcoxon rank-sum tests for continuous variables and chi-squared tests for categorical variables. We used logistic regression, comparing TR to DA cases, to examine the effect of adjusting for age for each covariate group. Recovery time was calculated as symptom end date minus symptom onset date. For cases whose symptoms were on-going at the time of interview, a minimum recovery period was calculated as the interview date minus the symptom onset date.

### *Salmonella* Enteritidis (SE) risks associated with travel

The Statistics Canada International Travel Survey was used as a source of denominator data for travel patterns for Ontario residents. The year 2009 was the most recent complete year of data available from this survey at the time of analysis [20]. Respondents of this survey were obtained from a simple random sample of the total number of travellers through port of entry by yearly quarter. We extracted information from 7,826 interviews, accounting for 16,870 travellers, for this analysis. Data on principal country of travel, age, gender, travel start-date and travel end-date were used for this analysis. Data on illness were not available in this dataset. For respondents visiting multiple countries during one trip, we used the first country where the respondent had spent two or more nights as the principal country, excluding those that did not spend at least two nights in a single destination abroad. We excluded those who returned from their travels during time intervals where case data were not collected (i.e., November 16 to January 7). Using a weighting system developed by Statistics Canada for the International Travel Survey, we determined the estimated number of international travellers among Ontario residents. For regression analyses, we included only those travellers with a principal travel country other than the USA (1,708 interviews, representing 3,710 travellers) as “controls”. The USA was excluded from these analyses since there were only two TR SE cases that travelled to the USA, and inclusion of this small number would have yielded unstable regression parameter error estimates.

We developed two multivariate logistic regression models using a manual, forward stepwise selection approach including variables significant at the P ≤ 0.05 level. We designed our logistic regression analysis based on the study conducted by Ekdahl et al. [15]. We used TR SE as cases and International Travel Survey travellers as “controls” to estimate the odds of infection in 1) different world regions and in 2) Caribbean countries and Mexico. We tested age, sex and travel period (summer vs. winter) for possible confounding and effect modification in both models. The final models presented include only statistically significant (P ≤ 0.05) covariates of the risk of acquiring SE from travel to a particular region or country.

Data analyses were conducted using SAS version 9.2 (SAS Institute Inc., Cary, NC, USA), STATA version 10.1 (Statacorp, TX, USA) and SPSS version 19 (SPSS Inc., an IBM Company).

## Results

There were a total of 998 laboratory-confirmed cases during the study period. Of the 998 cases, 165 (16.5%) cases met our exclusion criteria, leaving 833 cases. Of the 833 cases, 209 (25.1%) were either lost to follow-up or refused to participate, resulting in 624 cases included in this analysis. Thus, the response rate for this study was calculated as 74.9% (624/833). The response rate varied by investigation stage (Stage 1: 62.0% (204/329); Stage 2: 83.3% (420/504)). Cases lost to follow-up and refusals compared to those included in the analysis did not differ with respect to age (P = 0.105), however, cases lost to follow-up and refusals were more likely to be male (P = 0.035) and identified during Stage 1 of the study collection period (P < 0.0001).

**Figure 1:**
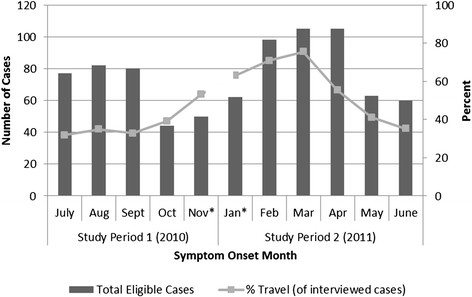
***Salmonella*Enteritidis infections, by symptom onset month and percent travel-related July 2010 to June 2011.** *Data are incomplete for November 2010 and January 2011, given the study period start and end dates. The total number of eligible cases includes both interviewed cases (n = 624) and cases lost to follow-up (n = 202). Symptom onset dates are estimated for lost to follow-up cases based on the mean delay (12 days) from symptom onset date to laboratory received date for interviewed cases. The percent travel cases by month was calculated by dividing the number of TR cases by the number of interviewed cases per month.

Of the 624 cases in this study, 324 (51.9%) were TR cases and 300 (48.1%) were DA. Figure 1 presents the number of eligible cases and the proportion of interviewed SE cases in a given month that were TR. In the winter travel period (November, and January to April), the proportion of TR cases was 65.1%, while in the summer travel period (May-October), 35.7% of cases were TR.

### Comparison of travel-related (TR) and domestically-acquired (DA) *Salmonella* Enteritidis (SE) cases

The illness characteristics of TR and DA cases, examined via symptoms and recovery time revealed one significant difference: a higher proportion of TR cases reported experiencing nausea than DA cases (66.4% vs. 51.7%, P < 0.001), which remained significant after adjusting for age (OR 1.86, 95% CI 1.25 to 2.50). All other symptoms were reported in similar proportions for TR and DA cases. While TR cases reported a longer minimum recovery time (11 days vs. 9 days, P = 0.004), this difference did not persist after adjusting for age (OR 1.02, 95% CI 0.99 to 1.04).

The demographic characteristics and health-seeking behaviour of TR and DA cases are presented in Table 1. TR cases were older, with a median age of 35 years compared to 21 years among DA cases (P < 0.001). Significantly fewer TR cases visited the Emergency Room and were admitted to the hospital than DA cases (P = 0.003 and P < 0.001, respectively). Both of these associations were independent of age (OR 0.58, 95% CI 0.41 to 0.82 and OR 0.31, 95% CI 0.16 to 0.57, respectively).

**Table 1:** **Demographic characteristics and health-seeking behaviour of domestically-acquired (n = 300) and travel-related (n = 324) cases of*Salmonella*Enteritidis**

	**Travel**		**Domestic**		**Total**		P value*
	**n**	**%**	**n**	**%**	**n**	**%**	
**Age (median)**							
Median (yrs)	35		21				<0.001
0–11	51	*15.7*	93	*31.0*	144	*23.1*	
12–19	25	*7.7*	47	*15.7*	72	*11.5*	
20–34	83	*25.6*	64	*21.3*	147	*23.6*	
35–54	105	*32.4*	54	*18.0*	159	*25.5*	
55–64	40	*12.3*	17	*5.7*	57	*9.1*	
65+	20	*6.2*	25	*8.3*	45	*7.2*	
Male**	175	*54.2*	153	*51.3*	328	*52.6*	0.479
**Symptoms**^**§**^							
Diarrhea	320	*99.4*	291	*98.0*	611	*98.7*	0.124
Abdominal Cramps	280	*88.3*	252	*86.9*	532	*87.6*	0.686
Nausea	213	*66.4*	152	*51.7*	365	*59.4*	<0.001
Vomiting	127	*39.7*	116	*39.3*	243	*39.5*	0.926
Fever	219	*68.4*	217	*73.1*	436	*70.7*	0.523
Other	214	*68.2*	183	*62.5*	397	*65.4*	0.140
**Visited Emergency Room†**	105	*33.4*	131	*45.2*	236	*36.8*	0.003
**Admitted to hospital**‡	16	*5.1*	45	*15.2*	61	*10.0*	<0.001

*Chi-squared test p-value; **Three cases missing sex (2 DA, 1 TR). § Five cases were missing diarrhea (3 DA, 2 TR) data, 17 abdominal cramps (10 DA, 7 TR), 9 nausea (6 DA, 3 TR), 9 vomiting (5 DA, 4 TR), 7 fever (3 DA, 4 TR); and 17 other (7DA, 10TR). † Twenty cases missing emergency-room information (10 TR, 10 DA); ‡Fourteen cases missing admission to hospital information (11 TR, 3 DA).

### *Salmonella* Enteritidis (SE) risks associated with travel to various world regions and countries

Two cases were excluded as they had reported visiting multiple world regions. Of the remaining 322 TR cases, 283 (88.9%) were acquired in the Caribbean and Mexico region (Table 2). Of these 283 cases, 268 (94.7%) travelled to five countries: Dominican Republic, Cuba, Jamaica, Mexico and Antigua. Further, of the 283 cases that travelled to this region, 255 (90.1%) stayed on a resort. The median travel duration was 7.0 days (Inter-quartile range 7.0 to 8.0).

**Table 2:** **Distribution of travel-related*Salmonella*Enteritidis cases by world region, and by Caribbean/Mexico region**

**Travel region/country**	**Estimated no. of travelers†**	**Controls†**	**Reported cases††**	**Rate/100 000**
Africa	108,486	75	6	5.53
Asia	419,489	449	15	3.58
Caribbean and Mexico	1,173,785	1,682	283	24.11
*Antigua*	*17,433*	*16*	*13*	*74.57*
*Cuba*	*287,797*	*418*	*77*	*26.75*
*Dominican Republic*	*370,653*	*549*	*108*	*29.14*
*Jamaica*	*57,465*	*78*	*53*	*92.23*
*Mexico*	*254,160*	*418*	*17*	*6.69*
Other*	*186,277*	*203*	*15*	*8.05*
Central America	85,476	112	4	4.68
Europe	1,267,932	1,240	8	0.63
Oceania	26,521	52	1	3.77
South America	106,769	100	3	2.81
USA	5,920,574	13,160	2	0.03
**Total**	**9,109,032**	**16,870**	**322**	**3.53**

† Number of travellers is estimated based on the number of survey respondents (controls) from the 2009 Statistics Canada International Travel Survey.

††Two cases (0.6%) were excluded from this analysis because they had visited multiple world regions.

*“Other” includes Bahamas, Barbados. Bermuda, Cayman Islands, St. Kitts, St. Lucia, St. Vincent, Trinidad & Tobago, Turks & Caicos, British Virgin Islands, Dutch West Indies, Guadeloupe, Haiti, Puerto Rico, US Virgin Islands, and West Indies N.E.S.

The lowest risk of TR SE cases per 100,000 was for travellers to the USA (0.03) and Europe (0.63) and the highest risk was for travellers to the Caribbean and Mexico Region (24.11) (Table 2). The highest odds of being a TR SE case was for those who had travelled to the Caribbean (OR 37.29, 95% CI 17.87 to 77.82) when controlling for the effect of age and the travel period compared to cases who had travelled to Europe (Table 3). In the Caribbean/Mexico region, Antigua, Jamaica and Barbados had the highest odds ratios, OR 19.63 (95% CI 7.78 to 49.52), 17.28 (95% CI 9.29 to 32.13) and 13.18 (95% CI 2.96 to 58.69), respectively, compared to Mexico when adjusting for age and travel period.

**Table 3:** **Odds of being a travel-related case of*Salmonella*Enteritidis by travel destination**

	**Unadjusted**		Adjusted*	
	**OR**	**95% CI**	**OR**	**95% CI**
**World Region Analysis (Cases: 318, Controls: 3,454)†**				
**Region**				
*Europe*	Ref	*-*	Ref	*-*
*Africa*	12.40	*4.20–36.66*	14.01	*4.68–41.91*
*Asia*	5.18	*2.18–12.30*	6.41	*2.67–15.41*
*Central America*	5.54	*1.64–18.67*	5.85	*1.71–20.03*
*Caribbean*	32.37	*15.95–65.70*	37.29	*17.87–77.82*
*North America (Mexico only)*	6.30	*2.70–14.71*	6.89	*2.86–16.60*
*Oceania*	2.98	*0.37–24.28*	3.68	*0.45–30.25*
*South America*	4.65	*1.22–17.80*	5.71	*1.48–22.08*
**Age (years)**				
0–11	9.53	*5.50–16.49*	7.85	*4.37–14.13*
12–19	3.22	*1.75–5.92*	2.36	*1.25–4.46*
20–34	3.60	*2.18–5.96*	2.31	*1.37–3.90*
35–54	2.32	*1.42–3.79*	1.61	*0.97–2.66*
55–64	1.31	*0.75–2.29*	1.08	*0.61–1.91*
65+	Ref	*-*	Ref	*-*
**Travel Period:** “Winter”	0.50	*0.39 – 0.65*	1.31	*0.98–1.74*
**Caribbean Country and Mexico Sub-Analysis (Cases: 279, Controls: 1,489)‡**				
**Country**				
*Mexico*	Ref	*-*	Ref	*-*
*Cuba*	4.53	*2.63–7.79*	4.75	*2.73–8.25*
*Dominican Republic*	4.84	*2.86–8.19*	5.00	*2.92–8.56*
*Jamaica*	16.71	*9.19–30.36*	17.28	*9.29–32.13*
*Antigua*	19.98	*8.30–48.07*	19.63	*7.78–49.52*
*Curacao*	1.12	*0.25–5.00*	1.50	*0.33–6.91*
*Bahamas*	2.20	*0.78–6.18*	2.02	*0.70–5.85*
*Barbados*	12.29	*2.83–53.38*	13.18	*2.96–58.69*
*St. Lucia*	8.20	*0.81–82.95*	6.59	*0.64–68.44*
**Age (years)**				
0–11	5.35	*2.91–9.83*	5.75	*3.02–10.95*
12–19	2.10	*1.08–4.08*	2.39	*1.20–4.77*
20–34	2.12	*1.23–3.63*	1.78	*1.01–3.14*
35–54	1.42	*0.84–2.39*	1.28	*0.74–2.20*
55–64	0.80	*0.44–1.48*	0.80	*0.43–1.50*
65+	Ref	*–*	Ref	*–*
**Travel Period:** “Winter”	1.83	*1.36–2.46*	1.35	*0.96–1.89*

*The adjusted odd ratios (ORs) presented are each adjusted for all of the other significant variables in the model (i.e. age category, travel period, and region or country).

† Of the total 324 cases, there were 6 cases excluded because of the following: two visited multiple world regions, two travelled to the USA, and two visited multiple countries within the Caribbean; of the total 3,710 controls, 256 were excluded because of missing age.

‡ Of the total 283 cases who travelled to the Caribbean and Mexico, 4 cases were excluded because of the following: two visited multiple countries within the Caribbean, and two cases were excluded because they travelled to Grenada and there were no controls who had travelled to Grenada; of the total 1,682 controls who travelled to the Caribbean and Mexico, 99 were excluded because of missing age, and 94 were excluded because no cases visited their respective countries: Bermuda (6), St. Kitts (4), St. Vincent (3), Trinidad & Tobago (29), Turks & Caicos Is. (1), British Virgin Islands (1), Guadeloupe (29), Haiti (1), Puerto Rico (15), West Indies, N.E.S. (5).

### Travel characteristics and phage type (PT) distributions by common travel destination among travel-related cases

**Figure 2:**
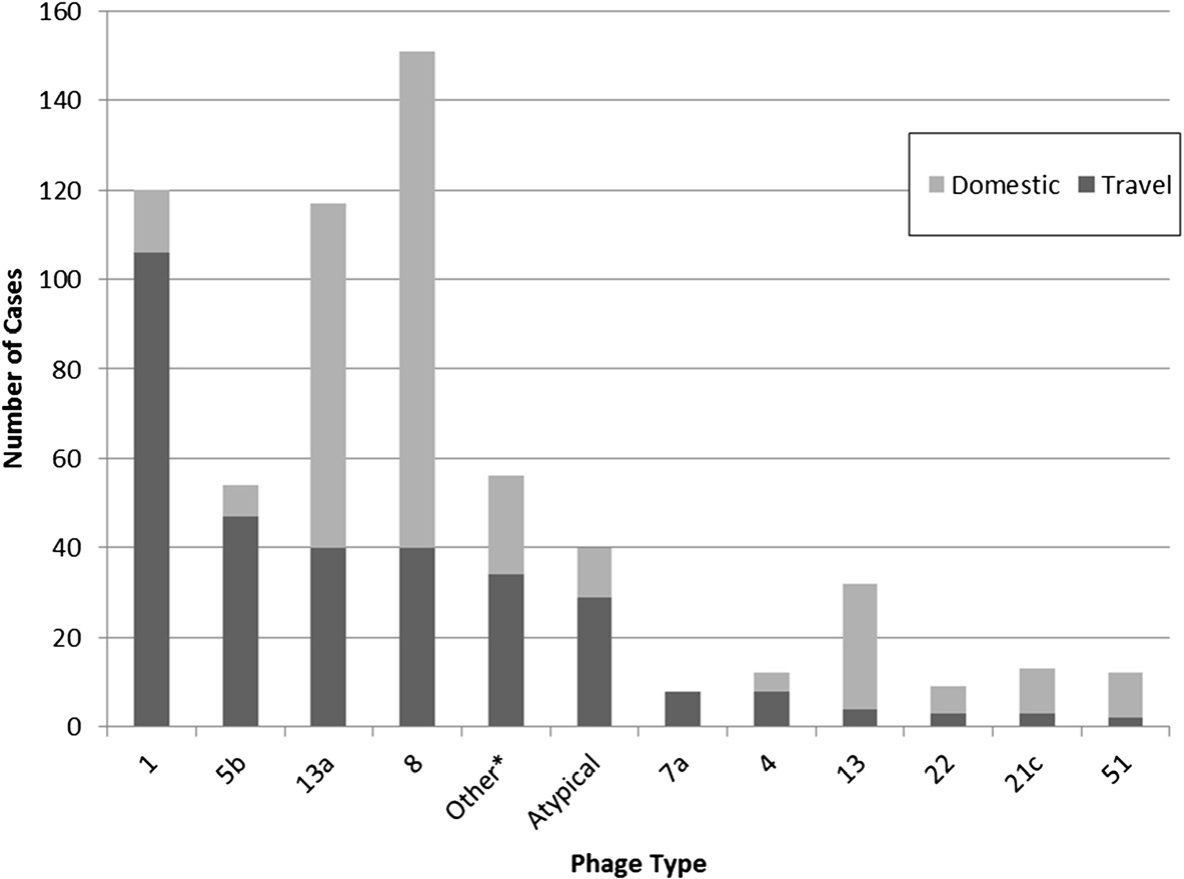
**Comparison of phage types between domestically-acquired (n = 300) and travel-related (n = 324) cases of*Salmonella*Enteritidis.** *Other phage types include those with 7 or fewer cases.

The distribution of PTs for SE cases by travel status is shown in Figure 2. PTs 8, 1 and 13a were the three most frequently detected PTs during our study period, accounting for 62.2% of all cases. The majority of cases with PTs 7a, 1, 5b, Atypical and 4 reported travel exposure, while cases with PTs 8, 13a, 13, 22, 21c, and 51 were predominantly DA.

Examination of the PT distributions from TR cases returning from the five most commonly visited countries revealed a distinct PT distribution (Table 4). PT 1 accounted for 88.9% of the cases who travelled to the Dominican Republic, PT 5b accounted for 58.4% of the cases returning from Cuba, PT 7a accounted for 47.1% of the cases returning from Mexico, and PT 13a and 8 were the two most common PTs isolated from travellers to Jamaica (56.6% PT 13a and 35.8% PT 8) and Antigua (46.2% PT 8 and 53.8% PT 13a).

**Table 4:** **Distribution of phage types for the five most common travel destinations among travel-related*Salmonella*Enteritidis cases**

**Country and phage type**	2010*		2011**		**Total**	
	**n**	**%**	**n**	**%**	**n**	**%**
**Antigua**						
*13a*	0	0.0	6	46.2	6	46.2
*8*	0	0.0	7	53.8	7	53.8
*Total*	0	*-*	13	*100.0*	13	*100.0*
**Cuba**						
4	2	8.3	2	3.8	4	5.2
5b	18	75.0	27	50.9	45	58.4
8	1	4.2	2	3.8	3	3.9
Atypical	2	8.3	21	39.6	23	29.9
Other^1^	1	4.2	1	1.9	2	2.6
*Total*	*24*	*100.0*	*53*	*100.0*	*77*	*100.0*
**Dominican Republic**						
1	14	100.0	82	87.2	96	88.9
53	0	0	2	2.1	2	1.9
5b	0	0	2	2.1	2	1.9
8	0	0	3	3.2	3	2.8
Other^2^	0	0	5	5.3	5	4.6
*Total*	14	*100.0*	94	*100.0*	108	*100.0*
**Jamaica**						
13a	5	50.0	25	58.1	30	56.6
8	5	50.0	14	32.6	19	35.8
2	0	0.0	2	4.7	2	3.8
Other^3^	0	0.0	2	4.7	2	3.8
*Total*	*10*	*100.0*	*43*	*100.0*	*53*	*100.0*
**Mexico**						
7a	2	66.7	6	42.9	8	47.1
21	0	0.0	2	14.3	2	11.8
Other^4^	1	33.3	6	42.9	7	41.2
*Total*	3	*100.0*	14	*100.0*	17	*100.0*

* - specimen collection date July 11 to November 30, 2010.

** - specimen collection date January 20 to July 31, 2011.

Other phage types were grouped if only one positive case for that phage type was recorded: ^1^: PTs 27 and 29a; ^2^: PTs 1b, 4, 6a, 13, and 37; ^3^: PTs 22 and 51; ^4^: PTs 1, 6, 6a, 8, 18, 22, and 41.

## **Discussion**

During the investigation period, 51.9% of SE cases in Ontario were TR. This finding was surprising to us as unpublished Reportable Disease data by Vrbova et. al., indicated that the proportion of TR cases for salmonellosis was approximately 20%. This finding was consistent, however, with a 2010 publication of findings from a sub-population of Ontario. Of the SE cases identified in the Region of Waterloo, 48.7% were classified as TR for the period June 2005 - May 2009 [11]. In our study, the TR SE cases occurred predominately in the winter months when more Ontario residents travel to “sun” destinations. Previously, similar findings pertaining to TR cases and seasonality were found in the province of British Columbia [12]. Separating TR and DA cases in analyses of SE surveillance data facilitates identifying relevant trends and potential clusters for investigation.

The number of SE cases in Ontario was 1,035 in 2010 and the estimated population was 13,227,800 [9,16]. Thus, the rate of SE cases in Ontario in 2010 was 7.82 per 100,000 persons. The rates of SE per 100,000 travellers for the Caribbean and Mexico region (24.11), Antigua (74.57), Cuba (26.75), Dominican Republic (29.14), and Jamaica (92.23) were markedly higher than the overall rate for Ontario. In contrast, the SE rates for travellers to the USA (0.03) and Europe (0.63) were lower than the overall rate for Ontario. Regression analysis revealed some statistically significant findings. The odds of acquiring SE for travellers to the Caribbean were 37 times greater than for travellers to Europe. Further, the odds of acquiring SE in the Caribbean were significantly higher than the odds of acquiring SE for travellers to Asia and Mexico. Among travellers to the Caribbean and Mexico region specifically, there were five countries (i.e., Antigua, Jamaica, Barbados, Dominican Republic, and Cuba) for which the odds of acquiring SE were significantly higher than for Mexico. The degree to which the rates in the various travel destinations change from year to year is not known. Nonetheless, having a good understanding of the rates in the travellers to different parts of the world would assist those providing advice to prospective travellers to those regions with a higher risk of acquiring illness. Freedman et. al., found that only 30% of travellers to the Caribbean sought pre-travel medical advice which was less than the 55% of travellers seeking advice for travelling to other developing regions [21]. This might indicate that travellers believe the health risks associated with the Caribbean are less than those associated with other travel destinations in the developing world.

The Caribbean countries and Mexico are well known winter travel destinations for Ontario residents, in part, because of the numerous “all-inclusive” resorts. For this reason, Mexico was grouped with the Caribbean *a priori*. Our data showed that 88.9% of reported TR illnesses were acquired in this region. Further, our data revealed that 90.1% of all SE cases travelling to the Caribbean and Mexico region had stayed at a resort. Given the high percentage of SE cases that travelled to the Caribbean and who stayed at a resort, further investigation should be considered in regard to the role that these resorts have in being a source of illness. While our investigation only considered SE, further investigation should also be considered in regard to the role of the Caribbean and resorts for other enteric pathogens. A recent study of a Canadian community by Ravel et. al., reported that 25% of *Campylobacter*, 13% of *Giardia*, 66% of non-typhoidal *Salmonella*, 44% of *Shigella*, and 89% of Yersiniosis TR cases stayed at a resort [11].

Identifying both the *Salmonella* serotypes and PTs can be instrumental for identifying sources of infection for sporadic and outbreak cases [22,23]. In our investigation, we also made use of the PT findings by making associations with the travel status of a case. In Ontario, prior to this investigation, there were three PTs that were thought to be most frequently DA (i.e., PT 8, 13 and 13a). Other PTs were thought to be most frequently TR (i.e., 1, 4, and 5b). There were other PTs, especially newly identified and infrequently identified PTs, for which the TR or DA association was not well understood (e.g., Atypical, 21c, 51). The investigation improved our knowledge of the association between travel and PTs. We learned that there were a higher percentage of TR PT 8 and PT 13a cases than previously thought, although these PTs remain predominantly DA, and that these TR cases occurred primarily in the winter consistent with the winter travel period. We also confirmed our understanding that PTs, 1, 4, and 5b were TR although we did learn that there was a small percentage of these PTs that were acquired in Ontario. Improvements in the quality of Reportable Disease data would assist with improving the capability to assess the TR status of the various PTs.

Our investigation also revealed that there were distinct associations between various PTs and the five most frequently visited countries, i.e., Antigua and Jamaica – PT 8 and 13, Cuba – PT 5b, Dominican Republic – PT 1, and Mexico PT 7a. It is not known whether these associations are consistent from year to year. Further investigation in regard to the PT trends over time from the various countries is warranted. These associations may assist with identifying the source of the illnesses, possibly in regard to the consumption of imported and domestically obtained food items.

### Limitations

The purpose of the original study was to identify the source of the increase of SE in Ontario using a hypothesis-generating stage to inform the subsequent case–control study. Thus, the original study design was not intended to consider detailed characteristics of TR cases. Further, for logistical reasons, in the transition between the hypothesis-generating stage and the case–control stage, case interviews were discontinued from December 1, 2010 to January 19, 2011. Certainly, having the data for this omitted period would have been useful for continuity.

The definition of a TR case was a person who travelled outside of Canada within the three days prior to onset of illness. Misclassification of TR cases may occur with this definition resulting in an over-estimation of TR illness. For example, cases with onset of illness soon after departure, or cases with illness onsets two or three days after return, may have acquired their illness in Ontario. If we reclassified the nine cases in this study who had a symptom onset date within one day of travel departure and the 44 cases with illness onsets two or three days following their return, the proportion of travel-related cases would decrease by less than 10% from 51.9% to 43.4%.

Data on illness was not collected on the traveller respondents in the Statistics Canada International Travel Survey. Therefore, it is possible that this control group could include some cases. However, using the highest rate we found in travellers, we would expect three cases at most to have been misclassified as controls, and having included them in our analysis would have biased our results toward the null. Another limitation was that controls for the various travel destinations were obtained from 2009 data, since 2010 and 2011 data were not available at the time of writing. While it is unlikely that large changes in travel patterns occurred between 2009 and 2010–11, such changes would impact both our rate calculations and our logistic regression results, especially for smaller destinations in the Caribbean.

## Conclusion

Our investigation into the source of an increase in SE cases in Ontario led to the finding that TR cases represent a considerable burden of SE illness in Ontario. A large majority of the TR SE cases were acquired while staying at a resort in the Caribbean and Mexico region. For Ontario travellers to various destinations in the world, the odds of acquiring SE were the highest for the Caribbean.

Collectively, these findings would prove useful to other jurisdictions with travel patterns in their residents similar to Ontario residents. Having a good understanding of whether cases were TR or DA is useful for understanding the source and risk of infections as well as trends in surveillance data. The findings would also be useful for those involved with providing health advice to travellers to the Caribbean. Finally, the travel destinations identified that were associated with contracting SE contribute to a better understanding of the global epidemiology of SE.

## Abbreviations

DA = Domestically acquired; PT = Phage type; SE = Salmonella Enteritidis; TR = Travel related; USA = United States of America.

## Competing interests

The authors declare that they have no competing interests.

## Authors’ contributions

DM, CV and YW conceived of the study. MKT, RS, LV, RW, CV, BL, YW and DM developed the study design and methodology. MKT, RS, RW, and CJ were involved with interviewing cases. MKT, RS, MT, LV, and BD were involved with data management and analysis. VA, AM, and RA were involved in laboratory analysis. MKT, RS, MT, LV, VA, RW, CV, BL, BD, NC, and DM were involved with drafting the manuscript. All authors read and approved the final manuscript.

## Pre-publication history

The pre-publication history for this paper can be accessed here:

http://www.biomedcentral.com/1471-2458/12/310/prepub
